# Quantitative measurement of vascular density and flow using optical coherence tomography angiography (OCTA) in patients with central retinal vein occlusion: Can OCTA help in distinguishing ischemic from non-ischemic type?

**DOI:** 10.1186/s40942-018-0152-9

**Published:** 2018-12-27

**Authors:** Alireza Khodabandeh, Kiyanoosh Shahraki, Ramak Roohipoor, Hamid Riazi-Esfahani, Mehdi Yaseri, Houshang Faghihi, Fatemeh Bazvand

**Affiliations:** Eye Research Center, Farabi Eye Hospital, Tehran University of Medical Science, Qazvin Square, 1336616351 Tehran, Iran

**Keywords:** Central retinal vein occlusion, Ischemic, Non-ischemic, Optical coherence tomography angiography

## Abstract

**Background:**

To evaluate microvascular changes and quantitative parameters in patients with central retinal vein occlusion (CRVO) by using optical coherence tomography angiography (OCTA) and finding difference between presumably ischemic and non ischemic CRVO.

**Methods:**

Patients with CRVO (31) and healthy control (20) were enrolled in this observational case control study. The OCTA was done for each patient and control subject. In macular area 2 images were taken for each eye (3 × 3 mm and 8 × 8 mm). The images were analyzed at three capillary plexuses (superficial and deep retinal capillary layers and choriocapillaris layer).

**Results:**

Thirty-one patients with CRVO (mean age 60.00 ± 13.72 years) and 20 healthy age/gender matched subjects (mean age 54.10 ± 12.33 years) were enrolled in this study (p = 0.095). The mean visual acuity of patients was 0.47 ± 0.54 LogMAR. Eyes with CRVO as compared with fellow eyes and control group showed significant reduction of flow in superficial (1.171 ± 0.262 vs. 1.362 ± 0.285 vs. 1.453 ± 0.105) and deep capillary plexus (1.042 ± 0.402 vs. 1.331 ± 0.315 vs. 1.526 ± 0.123) and choriocapillaris (1.206 ± 0.543 vs. 1.841 ± 0.308 vs. 1.966 ± 0.05) and vascular density in superficial (45.92 ± 4.2 vs. 50.99 ± 4.35 vs. 52.85 ± 2.99) and deep (48.03 ± 4.71 vs. 55.86 ± 3.81 vs. 58.2 ± 2.65) capillary plexuses. Some parameters (flow of both retinal capillary plexuses and parafoveal vascular density in deep plexus) showed significantly reduction in fellow eyes than control group. The parameters including flow [superficial (1.014 ± 0.264 vs. 1.279 ± 0.19) and deep (0.873 ± 0.442 vs. 1.152 ± 0.32) capillary plexuses and choriocapillaris (0.79 ± 0.327 vs. 1.424 ± 0.51)] and vascular density [superficial (44.24 ± 2.13 vs. 46.58 ± 4.13) and deep (45.28 ± 3.5 vs. 49.32 ± 3.94) capillary plexuses] were lower significantly in ischemic type than non ischemic CRVO. The most damaged parameter was flow in deep capillary plexus. The model with smallest Akaike information criterion and Bayesian information criterion was chosen as the best model. For easier calculation, we also calculated the reduced model. By choosing the threshold of 12.6, the formula [3.9 × F_1S_ + 0.8 × F_3S_] can diagnose the presumably ischemic CRVO from non ischemic type with AUC of 0.84, sensitivity of 100% and specificity of 69%. (F_1S_: flow in the central 1 mm-radius-circle of superficial plexus and F_3S_: flow in the central 3 mm-radius-circle of superficial plexus).

**Conclusion and relevance:**

In CRVO patients, the OCTA can accurately evaluate changes in microvascular structures. It may help in differentiation ischemic CRVO from non-ischemic CRVO.

## Introduction

Central retinal vein occlusion (CRVO) is a second common cause of retinal vascular disease after diabetic retinopathy [1]. The assessment of capillary alternations by fluorescein angiography is limited in acute phase of CRVO because of edema and hemorrhage [2]. Optical coherence tomography angiography (OCTA) recently has been introduced as a non-invasive method providing micro-vascular assessment by using blood cell movement as natural contrast [3, 4].

There are some reports about the precise information provided by OCTA in retinal vein occlusion [5–8]. The accurate assessment of micro vascular anatomy was provided by using OCTA and was not possible by using fluorescein angiography. The assessment of capillary alternations by fluorescein angiography is limited in acute phase of CRVO because of edema and hemorrhage [9].

This study was designed to evaluate quantitative parameters using OCTA in patients with CRVO. Another goal of this study was to assess the difference of quantitative OCTA parameters in eyes with ischemic and non ischemic CRVO.

## Methods

**Fig. 1:**
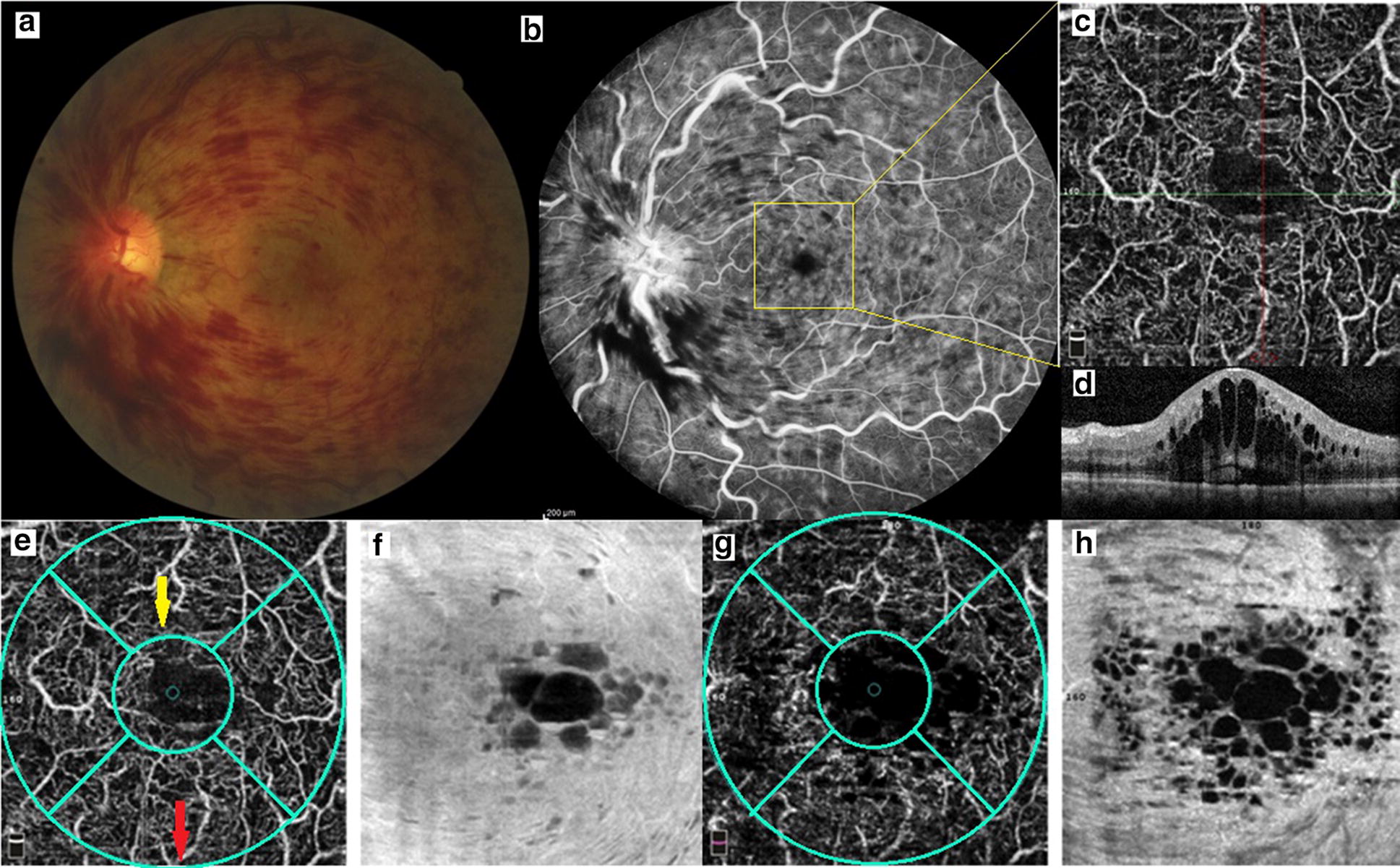
A patient with central retinal vein occlusion. **a** Fundus photograph, **b** fluorescein angiography, **c** optical coherence tomography angiography (OCTA) in superficial capillary plexus, **d** cystoids macular edema is observed in optical coherence tomography (OCT). **e**, **g** Whole vascular density (larger green circle: red arrow), foveal vascular density (small green circle: yellow arrow) and parafoveal vascular density (difference between 2 circles) were calculated in superficial and deep capillary plexuses in OCTA. **f**, **h** En face OCT at the level of superficial and deep capillary plexuses showed cystoids macular edema

**Fig. 2:**
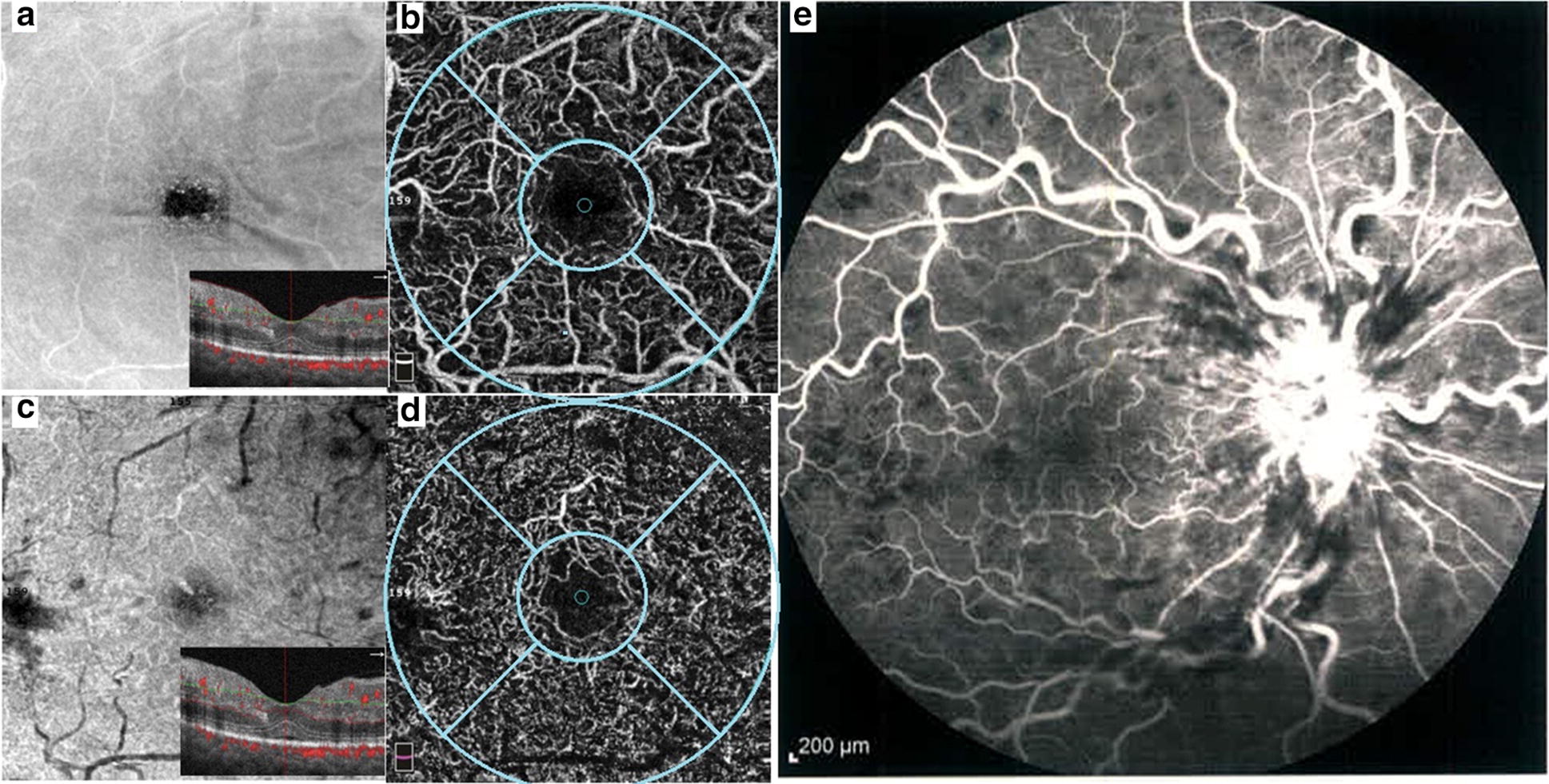
A patient with central retinal vein occlusion. **a** En face optical coherence tomography at superficial retina layer with segmentation lines of superficial capillary layer. **b** Optical coherence tomography angiography (OCTA) in superficial capillary plexus, whole vascular density (larger green circle), foveal vascular density (small green circle) and parafoveal vascular density (difference between 2 circles) were calculated in superficial capillary plexuses in OCTA. **c** En face optical coherence tomography at deep capillary layer with segmentation lines of this plexus. **d** OCTA in deep capillary plexus. **e** Fluorscein angiography

This observational case control study was performed at Farabi Eye Hospital, Tehran, Iran from April 2016 to January 2017. It was approved by the Institutional Review Board of Tehran University of medical sciences and followed the tents of Helsinki. All patients provided written informed consent for participation in the study. Patients with clinical diagnosis of new onset treatment naïve CRVO (≤ 1 month) were enrolled in this study. Patients with significant media opacity, low image quality (signal strength index < 50), severe cystoids macular edema interfering with accurate analysis of OCTA image, history of intraocular injection, presence of other ophthalmologic conditions (diabetic retinopathy and combined retinal artery and vein occlusion) were excluded. Presumably ischemic from non ischemic type of CRVO was differentiated according to presence of relative afferent pupillary reflex and visual acuity equal or less than 20/200. The right eyes of the healthy subjects with age and gender match were enrolled in the study as control group. Complete ophthalmic examination was done for all subjects. OCTA (Optovue, Inc, Fremont CA, USA) was done for all participants using the split spectrum amplitude decorrelation angiography algorithm. That instrument operated at 840 nm wavelength and did 70,000 A-scans per second to obtain OCTA. For each eye, 2 images were taken with the size of image scan 3 × 3 mm and 8 × 8 mm (macular area). The images were analyzed at three capillary layers consisting of superficial capillary network (by automated segmentation selecting area between internal limiting membrane and external boundary of ganglion cell layer), deep capillary network (by automated segmentation selecting area between inner plexiform layer and outer plexiform layer) and choriocapillaris (at 30 µm below the retinal pigment epithelium [RPE]). The segmentations were identified automated. Blood flow was calculated in 3 capillary networks inside the circle with 1 mm-radius and 3 mm-radius with the foveal center in the image 3 × 3 mm and 8 × 8 mm respectively. Vascular density was calculated as a percent of the area occupied by vessels in selected area and selected depth of vessels. Vascular density was determined in image 3 × 3 mm in superficial and deep capillary networks as followed label: foveal vascular density (vascular density within 1 mm circle of ETRDS), parafoveal vascular density (vascular density within 3 mm circle of ETDRS with subtraction of foveal vascular density) and whole vascular density (total vascular density within 3 mm circles of ETDRS). For further analysis the vascular density in parafoveal area was divided into 4 parts including nasal, superior, temporal and inferior (Figs. 1, 2).

## Statistical analysis

All data were analyzed by R (R Core Team (2014). R: A language and environment for statistical computing (R Foundation for Statistical Computing, Vienna, Austria. URL http://www.R-project.org/). p value of < 0.05 was accounted as statistically significant. Kolmogorov–Smirnov tests as well as Q–Q plot were used to confirm for the normal distribution of data. The flow and vascular density parameters of patients were compared with control group by using generalized estimating equation (GEE). Receiver-operator characteristic (ROC) curve analysis was assumed for each parameter. The areas under the ROC curve (AUROCs) were determined and by using Youden’s J statistics, the optimal threshold was obtained. The sensitivity, specificity, positive predictive value (PPV), negative predictive value (NPV) and the diagnostic accuracy levels for differentiation ischemic from non-ischemic type of CRVO and corresponding thresholds were evaluated for each parameter. The most damaged parameter was assessed by calculation of the ratio of each parameter in involved eye of patients to control average. Afterward, the parameter was selected according to repeated measure analysis of variance (Repeated measure ANOVA). The type 1 error was adjusted by using Bonferroni correction. We utilized logistic regression for obtaining the best combination of 12 parameters (2^12^ − 1 = 4095 models). The model with smallest AIC (Akaike information criterion) and BIC (Bayesian information criterion) was chosen as the best model. For easier calculation, we also calculated the reduced model.

## Results

Forty patients with CRVO and 20 healthy age/gender matched subjects were enrolled in this study. Nine patients were excluded because of poor image quality. There was insignificant difference (p = 0.095) between the mean age of patients (60.00 ± 13.72 years) and control subjects (54.10 ± 12.33). The proportion of male to female was 15/16 in patients and 5/15 in control subjects (Chi square 0.09). The mean visual acuity of patients was 0.47 ± 0.54 LogMAR (range 0.0–2.31). Patients with CRVO were divided into 2 groups; ischemic (13 patients with mean age 66.25 ± 11.04 years) and non-ischemic type (18 patients with mean age 46.88 ± 16.32 years).

Approximately all OCTA parameters including flow and vascular density were statistically significantly lower in affected eyes of patients as compared with fellow eyes or control group with exception of foveal vascular density in superficial and deep capillary plexuses (Table 1). Two latter parameters were significantly greater in involved eye of patients with CRVO as compared with fellow eye or control eyes.

**Table 1:** Vascular density and blood flow of the retinal capillary networks and choriocapillaris in eyes with central retinal vein occlusion (case) compared to fellow eyes of patients and healthy eyes (control)

	Groups Mean ± SD (range)			P§	P1	P2	P3
	Case	Fellow eye	Control				
Flow in superficial capillary network in 3 × 3 mm image	1.171 ± 0.262	1.362 ± 0.285	1.453 ± 0.105	< 0.001	0.003	0 < .001	0.183
	(0.426–1.583)	(0.455–1.949)	(1.159–1.625)				
Flow in deep capillary network in 3 × 3 mm image	1.042 ± 0.402	1.331 ± 0.315	1.526 ± 0.123	< 0.001	0.001	0 < .001	0.024
	(0.164–1.55)	(0.371–1.657)	(1.152–1.673)				
Flow in choriocapillaris in 3 × 3 mm image	1.206 ± 0.543	1.841 ± 0.308	1.966 ± 0.05	< 0.001	0 < .001	0 < .001	0.274
	(0.212–1.961)	(0.466–2.056)	(1.833–2.059)				
Whole vascular density of superficial capillary network	45.92 ± 4.2	50.99 ± 4.35	52.85 ± 2.99	< 0.001	0 < .001	0 < .001	0.069
	(38.93–57.95)	(39.24–57.34)	(47.59–57.57)				
Foveal vascular density of superficial capillary network	36.47 ± 9.78	30.13 ± 7.12	30.63 ± 6.44	< 0.001	0.003	0.018	0.824
	(22.53–65.34)	(19.4–54.78)	(13.29–42.25)				
Parafoveal vascular density of superficial capillary network	47.04 ± 5	52.91 ± 4.79	55.01 ± 3.45	< 0.001	0 < .001	0 < .001	0.062
	(37.3–61.39)	(41.27–58.92)	(48.02–59.5)				
Parafoveal vascular density of superficial capillary network in temporal area	44.75 ± 5.66	51.19 ± 6.11	53.7 ± 2.76	< 0.001	0 < .001	0 < .001	0.066
	(28.03–59.48)	(36.89–58.63)	(47.1–57.78)				
Parafoveal vascular density of superficial capillary network in superior area	47.37 ± 5.67	53.97 ± 4.7	55.76 ± 3.39	< 0.001	0 < .001	0 < .001	0.169
	(36.29–64.65)	(40.92–60.37)	(49.17–60.31)				
Parafoveal vascular density of superficial capillary network in nasal	48.09 ± 4.39	52.54 ± 5.03	53.92 ± 5.92	< 0.001	0.001	0 < .001	0.321
	(40.79–57.97)	(39.17–59.88)	(33.43–59.36)				
Parafoveal vascular density of superficial capillary network in inferior part	47.34 ± 7.08	53.52 ± 5.33	56.01 ± 3.89	< 0.001	0 < .001	0 < .001	0.169
	(32.56–63.46)	(40.75–60.45)	(48.54–61.06)				
Whole vascular density of deep capillary network	48.03 ± 4.71	55.86 ± 3.81	58.2 ± 2.65	< 0.001	0 < .001	0 < .001	0.857
	(39.8–59.3)	(46.59–60.07)	(52.2–63.1)				
Foveal vascular density of deep capillary network	34.15 ± 12.36	27.54 ± 6.44	28.99 ± 7.97	< 0.001	0 < .001	0 < .001	0.049
	(11.31–59.44)	(11.85–39.58)	(10.2–46.4)				
Parafoveal vascular density of deep capillary network	49.65 ± 5.4	58.35 ± 4.01	60.87 ± 3.55	< 0.001	0.005	0.045	0.559
	(37.89–62.66)	(48.55–63.47)	(50.87–68.2)				
Parafoveal vascular density of deep capillary network in temporal part	48.1 ± 6.59	55.92 ± 6.73	60.07 ± 3.18	< 0.001	0 < .001	0 < .001	0.011
	(32.27–61.94)	(36.15–64.19)	(53.44–67.18)				
Parafoveal vascular density of deep capillary network in superior part	49.94 ± 7.06	60.1 ± 4.82	62.53 ± 2.41	< 0.001	0 < .001	0 < .001	0.096
	(36.26–65.68)	(48.65–68.04)	(58.51–67.45)				
Parafoveal vascular density of deep capillary network in nasal part	50.88 ± 5.92	57.79 ± 5.36	58.52 ± 8.47	< 0.001	0 < .001	0.001	0.942
	(32.25–59.86)	(41.73–67.55)	(26.4–66.45)				
Parafoveal vascular density of deep capillary network in inferior part	49.05 ± 8.4	58.72 ± 5.09	62.75 ± 2.85	< 0.001	0 < .001	0 < .001	0.009
	(28.26–64.84)	(47.57–65.43)	(58.13–68.39)				
Flow in superficial capillary network in 8 × 8 mm image	10.952 ± 1.846	11.77 ± 1.497	12.591 ± 1.035	0.001	0.089	0 < .001	0.030
	(5.9–13.76)	(7.641–13.841)	(10.641–14.655)				
Flow in deep capillary network in 8 × 8 mm image	7.907 ± 3.155	10.449 ± 3.124	12.576 ± 1.742	< 0.001	0.002	0 < .001	0.004
	(1.452–13.215)	(3.517–14.988)	(9.874–15.157)				
Flow in choriocapillaris in 8 × 8 mm image	11.543 ± 4.802	16.757 ± 2.669	17.486 ± 0.414	< 0.001	0 < .001	0 < .001	0.392
	(1.443–17.378)	(2.828–17.987)	(16.393–18.344)				

Based on GEE, *SD* standard deviation; *P§* p value among three groups; *P1* p value between case and fellow eye; *P2* p value between case and control; *P3* p value between fellow eye and control

For obtaining further details, the analysis was performed between ischemic type of CRVO and non-ischemic CRVO. The results of this comparison were summarized in Table 2. The statistically significant reduction approximately in all parameters with exception of foveal vascular density in both superficial and deep capillary plexuses were found in eyes with presumably ischemic CRVO in comparison to eyes with non-ischemic CRVO. Also the reduction was not significant in parfoveal vascular density in superficial capillary plexus in nasal part and in deep capillary plexus in superior part. The results of ROC analysis for ischemic CRVO versus non-ischemic CRVO was shown in Table 3. The most AUC was belonged to flow in choriocapillaris of macular area (AUC = 0.889) followed by parafoveal vascular density of deep capillary plexus in superior part (AUC = 0.850).

**Table 2:** Vascular density and blood flow of the retinal capillary networks and choriocapillaris in eyes with ischemic central retinal vein occlusion (CRVO) compared to non-ischemic CRVO

	CRVO Mean ± SD (range)		Diff	95% CI		P§
	Non Ischemic	Ischemic		Lower	Upper	
Flow in superficial capillary network in 3 × 3 mm image	1.279 ± 0.19	1.014 ± 0.264	.264*	0.098	0.430	0.003
	(0.852–1.583)	(0.426–1.308)				
Flow in deep capillary network in 3 × 3 mm image	1.152 ± 0.32	0.873 ± 0.442	.282*	0.005	0.560	0.046
	(0.273–1.539)	(0.164–1.349)				
Flow in choriocapillaris in 3 × 3 mm image	1.424 ± 0.51	0.79 ± 0.327	.633*	0.304	0.962	0 < .001
	(0.431–1.931)	(0.212–1.431)				
Whole vascular density of superficial capillary network	46.58 ± 4.13	44.24 ± 2.13	2.542*	0.292	4.792	0.028
	(38.93–55.21)	(39.67–47)				
Foveal vascular density of superficial capillary network	33.77 ± 8.13	41.32 ± 10.77	− 7.955*	− 14.798	− 1.113	0.024
	(22.53–55.67)	(23.98–65.34)				
Parafoveal vascular density of superficial capillary network	48.09 ± 4.92	44.73 ± 2.11	3.385*	0.768	6.002	0.013
	(37.3–55.62)	(41.76–49.8)				
Parafoveal vascular density of superficial capillary network in temporal area	46.01 ± 5.67	42.34 ± 3.3	4.564*	1.550	7.578	0.005
	(28.03–53.26)	(36.55–48.08)				
Parafoveal vascular density of superficial capillary network in superior area	48.75 ± 5.16	44.72 ± 3.46	4.286*	0.957	7.615	0.013
	(36.29–54.99)	(38.56–52.2)				
Parafoveal vascular density of superficial capillary network in nasal	48.15 ± 3.86	47.3 ± 4.27	0.715	− 2.177	3.606	0.616
	(40.79–54.16)	(41.21–56.28)				
Parafoveal vascular density of superficial capillary network in inferior part	48.23 ± 7.19	44.8 ± 4.71	2.522	− 2.007	7.051	0.263
	(32.56–62.33)	(39.59–56.76)				
Whole vascular density of deep capillary network	49.32 ± 3.94	45.28 ± 3.5	4.023*	1.522	6.524	0.003
	(40.06–54.78)	(39.8–51.4)				
Foveal vascular density of deep capillary network	30.57 ± 10.61	38.88 ± 13.5	− 10.299*	− 18.585	− 2.012	0.017
	(11.31–53.33)	(19.63–59.44)				
Parafoveal vascular density of deep capillary network	51.18 ± 4.2	46.64 ± 4.54	4.340*	1.553	7.127	0.004
	(41.09–57.15)	(37.89–52.21)				
Parafoveal vascular density of deep capillary network in temporal part	48.45 ± 6.63	46.52 ± 5.24	7.167*	3.236	11.099	0.001
	(32.27–57.69)	(37.17–54.09)				
Parafoveal vascular density of deep capillary network in superior part	52.81 ± 6.19	45.5 ± 4.89	2.583	− 1.548	6.714	0.210
	(36.26–61.63)	(36.31–53.82)				
Parafoveal vascular density of deep capillary network in nasal part	51.81 ± 3.95	48.75 ± 7.49	6.218*	0.704	11.732	0.028
	(44.81–57.87)	(32.25–59.86)				
Parafoveal vascular density of deep capillary network in inferior part	51.04 ± 8.11	45.14 ± 6.31	1.769*	0.655	2.884	0.003
	(28.26–61.47)	(34.66–54.31)				
Flow in superficial capillary network in 8 × 8 mm image	11.677 ± 1.023	9.739 ± 2.043	3.253*	1.246	5.260	0.002
	(10.308–13.76)	(5.9–13.131)				
Flow in deep capillary network in 8 × 8 mm image	9.149 ± 2.178	5.965 ± 3.295	6.735*	3.921	9.549	0 < .001
	(6.061–13.215)	(1.452–10.58)				
Flow in choriocapillaris in 8 × 8 mm image	13.761 ± 3.853	7.383 ± 3.789	.282*	0.005	0.560	0.046
	(2.828–17.378)	(1.443–15.243)				

P§ based on GEE, *SD* standard deviation

**Table 3:** ROC results for ischemic central retinal vein occlusion (CRVO) versus non-ischemic CRVO

Parameter	AUC	95% CI	
		Lower	Upper
Flow in superficial capillary network in 3 × 3 mm image	0.825	0.681	0.969
Flow in deep capillary network in 3 × 3 mm image	0.709	0.527	0.892
Flow in choriocapillaris in 3 × 3 mm image	0.842	0.695	0.988
Whole vascular density of superficial capillary network	0.722	0.534	0.910
Foveal vascular density of superficial capillary network	0.731	0.546	0.916
Parafoveal vascular density of superficial capillary network	0.752	0.572	0.932
Parafoveal vascular density of superficial capillary network in temporal area	0.791	0.629	0.953
Parafoveal vascular density of superficial capillary network in superior area	0.756	0.581	0.931
Parafoveal vascular density of superficial capillary network in nasal	0.575	0.367	0.783
Parafoveal vascular density of superficial capillary network in inferior part	0.731	0.537	0.924
Whole vascular density of deep capillary network	0.803	0.641	0.965
Foveal vascular density of deep capillary network	0.679	0.468	0.891
Parafoveal vascular density of deep capillary network	0.795	0.637	0.953
Parafoveal vascular density of deep capillary network in temporal part	0.620	0.420	0.820
Parafoveal vascular density of deep capillary network in superior part	0.850	0.702	0.999
Parafoveal vascular density of deep capillary network in nasal part	0.624	0.406	0.842
Parafoveal vascular density of deep capillary network in inferior part	0.752	0.579	0.925
Flow in superficial capillary network in 8 × 8 mm image	0.803	0.624	0.983
Flow in deep capillary network in 8 × 8 mm image	0.748	0.565	0.931
Flow in choriocapillaris in 8 × 8 mm image	0.889	0.768	1.000

The correlation between visual acuity (LogMAR) and OCTA parameters was obtained by using Pearson correlation. The most significant direct correlation was observed between flow in choriocapillaris (in both image scan 8 × 8 mm and 3 × 3 mm) and visual acuity (r = − 0.802, p < 0.001 and r = − 0.745, p < 0.001 respectively). The direct correlation was obtained between visual acuity and all parameters with exception of inverse correlation of foveal vascular density in superficial (r = 0.415, p = 0.001) and deep (r = 0.458, p < 0.001) capillary plexuses with visual acuity. The visual acuity was directly correlated (with p value < 0.001) with the other parameters including whole vascular density in superficial (r = − 0.564) and deep (r = − 0.725) capillary plexuses, parafoveal vascular density in superficial (r = − 0.571) and deep (r = − 0.741) capillary plexuses, flow in superficial (r = − 0.471) and deep (r = − 0.530) capillary plexuses of macula.

## ROC curve

Best model: The discriminate score would be build base on

$$1100 \times {\text{F}}_{{1{\text{D}}}} - 3200 \times {\text{F}}_{{ 1 {\text{S}}}} - 20 \times {\text{VD}}_{\text{WD}} - 80 \times {\text{F}}_{{3{\text{CH}}}} \;{\text{formula}}.$$

(F_1D_: flow in the central 1 mm-radius-circle of deep plexus, F_1S_: flow in the central 1 mm-radius-circle of superficial plexus, VD_WD_: whole vascular density in deep plexus and F_3CH_: flow in the central 3 mm-radius-circle of choriocapillaris). This would have an AUC of 0.99 (95% CI 0.97–1), also the AIC of this model was 10. The best cutoff based on the Youden’s J index would be − 4300 with the sensitivity of 100% and specificity of 94%. The PPV and NPV for the model would be 90% and 100% respectively.

**Fig. 3:**
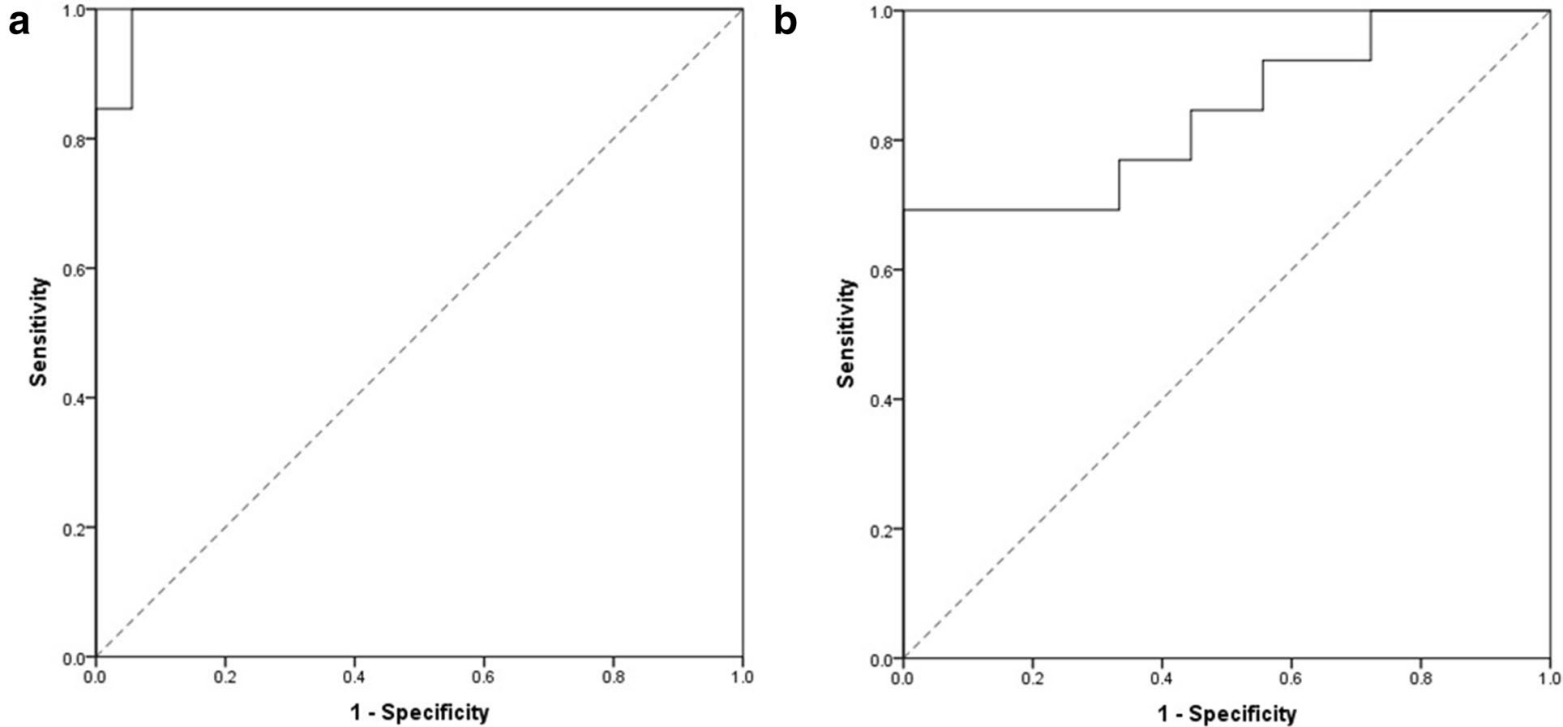
ROC curve of best model (**a**) and reduced formula (**b**) based on optical coherence tomography angiography parameters in distinguish ischemic central retinal vein occlusion (CRVO) from non ischemic CRVO

Reduced model: The discriminate score would be build base on

$$3.9 \times {\text{F}}_{{1{\text{S}}}} + 0.8 \times {\text{F}}_{{ 3 {\text{S}}}} \;{\text{formula}}.$$

(F_3S_: flow in the central 3 mm-radius-circle of superficial plexus). This would have an AUC of 0.84 (95% CI 0.70–0.99), also the AIC of this model was 34.6 the best cutoff based on the Youden’s J index would be 12.6 with the sensitivity of 100% and specificity of 69%. The PPV and NPV for the model would be 82% and 100% respectively (Fig. 3).

The mean ratios of all parameters are summarized in Table 4. The mean ratio of flow in deep capillary plexus of macular area was statistically lower than any other parameters (all p values were less than 0.042 based on Bonferroni correction method with exception for flow in choriocapillaris of macular area [p > 0.99], flow in deep plexus and choriocapillaris in 3 × 3 mm image [p > 0.99] and parafoveal vascular density in deep plexus [p = 0.544] which did not reach to statistically significant difference). In Ischemic and Non-Ischemic group, the mean ratio of case to average control was different in studied parameters (both p value < 0.001). There was no globally minimum mean ratio in parameter which is statistically significant compared to all other parameter (based on Bonferroni correction).

**Table 4:** Mean ratio of each parameter in involved eyes with central vein occlusion to their control average

	Total case		Non Ischemic CRVO		Ischemic CRVO	
	Mean ± SD	Median (IQR)	Mean ± SD	Median (IQR)	Mean ± SD	Median (IQR)
Flow in superficial capillary network in 3 × 3 mm image	0.8 ± 0.18	0.82 (0.69–0.94)	0.88 ± 0.13	0.93 (0.8–0.96)	0.7 ± 0.18	0.74 (0.65–0.81)
Flow in deep capillary network in 3 × 3 mm image	0.68 ± 0.26	0.75 (0.57–0.87)	0.75 ± 0.21	0.84 (0.65–0.9)	0.57 ± 0.29	0.67 (0.36–0.85)
Flow in choriocapillaris in 3 × 3 mm image	0.59 ± 0.27	0.5 (0.39–0.89)	0.72 ± 0.26	0.8 (0.52–0.94)	0.4 ± 0.17	0.45 (0.32–0.49)
Whole vascular density of superficial capillary network	0.86 ± 0.07	0.86 (0.82–0.91)	0.88 ± 0.08	0.9 (0.83–0.91)	0.84 ± 0.04	0.84 (0.82–0.86)
Foveal vascular density of superficial capillary network	1.21 ± 0.32	1.14 (0.98–1.42)	1.1 ± 0.27	1.02 (0.97–1.23)	1.35 ± 0.35	1.28 (1.14–1.53)
Parafoveal vascular density of superficial capillary network	0.85 ± 0.08	0.84 (0.8–0.91)	0.87 ± 0.09	0.9 (0.82–0.93)	0.81 ± 0.04	0.81 (0.8–0.84)
Parafoveal vascular density of superficial capillary network in temporal area	0.83 ± 0.09	0.85 (0.76–0.9)	0.86 ± 0.11	0.89 (0.82–0.91)	0.79 ± 0.06	0.78 (0.74–0.83)
Parafoveal vascular density of superficial capillary network in superior area	0.84 ± 0.09	0.84 (0.79–0.92)	0.87 ± 0.09	0.9 (0.8–0.95)	0.8 ± 0.06	0.81 (0.75–0.83)
Parafoveal vascular density of superficial capillary network in nasal	0.89 ± 0.07	0.89 (0.83–0.94)	0.89 ± 0.07	0.9 (0.86–0.95)	0.88 ± 0.08	0.87 (0.81–0.91)
Parafoveal vascular density of superficial capillary network in inferior part	0.84 ± 0.11	0.83 (0.76–0.9)	0.86 ± 0.13	0.88 (0.81–0.93)	0.8 ± 0.08	0.79 (0.74–0.81)
Whole vascular density of deep capillary network	0.82 ± 0.07	0.82 (0.77–0.87)	0.85 ± 0.07	0.86 (0.82–0.89)	0.78 ± 0.06	0.79 (0.72–0.81)
Foveal vascular density of deep capillary network	1.18 ± 0.43	1.1 (0.86–1.44)	1.05 ± 0.37	0.99 (0.86–1.11)	1.34 ± 0.47	1.3 (1.12–1.76)
Parafoveal vascular density of deep capillary network	0.81 ± 0.08	0.83 (0.76–0.86)	0.84 ± 0.07	0.84 (0.81–0.88)	0.77 ± 0.07	0.79 (0.7–0.82)
Parafoveal vascular density of deep capillary network in temporal part	0.79 ± 0.1	0.8 (0.74–0.87)	0.81 ± 0.11	0.83 (0.75–0.89)	0.77 ± 0.09	0.78 (0.69–0.84)
Parafoveal vascular density of deep capillary network in superior part	0.8 ± 0.11	0.82 (0.72–0.87)	0.84 ± 0.1	0.85 (0.82–0.92)	0.73 ± 0.08	0.74 (0.69–0.77)
Parafoveal vascular density of deep capillary network in nasal part	0.86 ± 0.1	0.86 (0.8–0.95)	0.89 ± 0.07	0.88 (0.84–0.95)	0.83 ± 0.13	0.82 (0.77–0.91)
Parafoveal vascular density of deep capillary network in inferior part	0.77 ± 0.13	0.8 (0.67–0.86)	0.81 ± 0.13	0.84 (0.76–0.92)	0.72 ± 0.1	0.73 (0.67–0.78)
Flow in superficial capillary network in 8 × 8 mm image	0.86 ± 0.14	0.87 (0.8–0.98)	0.93 ± 0.08	0.9 (0.86–0.99)	0.77 ± 0.16	0.77 (0.71–0.9)
Flow in deep capillary network in 8 × 8 mm image	0.62 ± 0.25	0.64 (0.48–0.76)	0.73 ± 0.17	0.66 (0.6–0.87)	0.47 ± 0.26	0.47 (0.23–0.71)
Flow in choriocapillaris in 8 × 8 mm image	0.63 ± 0.28	0.61 (0.5–0.88)	0.79 ± 0.22	0.87 (0.66–0.95)	0.42 ± 0.22	0.5 (0.23–0.52)

*SD* standard deviation; *CRVO* central retinal vein occlusion, *Mean* represents the mean ratio of each eye compared to the average of control group

## Discussion

In this study, we evaluated the quantitative measurement of vascular density and flow by using OCTA in patients with CRVO and compared them to healthy subjects. We found significant reduction in flow of three capillary networks including superficial and deep retinal capillary plexuses and choriocapillaris in affected eyes of patients. The decreasing parafoveal vascular density in superficial and deep capillary plexuses in eyes with CRVO was the other finding in this study. It can be partially explained by the presence of retinal vein occlusion that increases the intravascular pressure and hydrostatic pressure leading to decrease in flow [10]. Subsequently, the reduction in flow results in decreasing vascular density in OCTA that produce vascular image based on the existence of flow as natural contrast. The presence of macular edema might affect the flow and vascular density partly as a result of producing artifact. However, the reduction in retinal perfusion was documented in patients with retinal vein occlusion without cystoids edema [6]. So, this reduction is not explained only with artifact due to signal attenuation.

The other finding in this investigation was the reduction in flow of choriocapillaris. It may be explained by the mechanical pressure on the choriocapillaris flow owing to severe retinal edema and the presence of various inflammatory mediators may affect the choriocapillaris in addition to retinal vessels. The other probable explanation is that the artifact due to retinal edema produce false reduction in the choriocapillaris level due to masking effect [5]. Mastropasqua et al. [5] reported the decreased in vascular density in choriocapillaris in patients with retinal vein occlusion that it was improved after treatment in contrast to vascular density in superficial and deep capillary plexuses. This finding may support our theories especially the mechanical effect and probable artifact owing to edema in choriocapillaris level. The previous studies reported paradoxical findings about choroidal thickness in retinal vein occlusion including increasing and no significant alternation in choroidal thickness [11, 12]. The former one was explained with augmentation in VEGF and resultant vessel dilation of choroid. Finally, it can be concluded that the choroidal vessels may be influenced in retinal vein occlusion from these reports.

The other finding in this investigation was the reduction in flow of choriocapillaris. It may be explained by the mechanical pressure on the choriocapillaris flow owing to severe retinal edema and the presence of various inflammatory mediators may affect the choriocapillaris in addition to retinal vessels. The other probable explanation is that the artifact due to retinal edema produce false reduction in the choriocapillaris level due to masking effect [5]. Mastropasqua et al. [5] reported the decreased in vascular density in choriocapillaris in patients with retinal vein occlusion that it was improved after treatment in contrast to vascular density in superficial and deep capillary plexuses. This finding may support our theories especially the mechanical effect and probable artifact owing to edema in choriocapillaris level. The previous studies reported paradoxical findings about choroidal thickness in retinal vein occlusion including increasing and no significant alternation in choroidal thickness [11, 12]. The former one was explained with augmentation in VEGF and resultant vessel dilation of choroid. Finally, it can be concluded that the choroidal vessels may be influenced in retinal vein occlusion from these reports.

The foveal vascular density in both superficial and deep plexuses was significantly higher in eyes with CRVO than control eyes and fellow eyes of patients. We suggest this may be related to retinal edema and resultant disorganization of inner retinal layers leading to apparently increasing vascular density in foveal zone. Another explanation was the vascular engorgement in the foveal area that caused increasing vascular density in this area. No difference and reduction in superficial and deep plexus in foveal vascular density were founded in patients with CRVO in previous study by the Mastropasqua et al. [5]. This discrepancy may be related to difference in severity of CRVO in their patients with our patients.

Significant declines in macular flow in both superficial and deep capillary plexuses and parafoveal vascular density in deep plexus were observed in fellow eyes of our patients as compared to healthy subjects. The similar results were reported in previous study [6]. Adhi et al. [6] found reduction in vascular perfusion in affected eyes of their patients and also in 53% and 25% of fellow eyes of patients with CRVO and BRVO respectively. They also reported significant increase in foveal avascular zone in fellow eyes of their patients as compared to control group [6]. Previous reports recommended the increasing risk of vein occlusion in the fellow eyes of patients with retinal vein occlusion as compared with general population that showed compromised structure before vessel oclussion [13, 14]. It may be concluded that OCTA can detect earlier sign of occlusion and structure alternation in vulnerable patients. The further studies are required for confirming this hypothesis.

For further details we performed sub-analysis in eyes with CRVO. The flow and parafoveal vascular density were significantly lower in eyes with ischemic CRVO as compared with non ischemic CRVO. It can be concluded that more severity of ischemia related to more reduction in these parameters. Previous studies reported correlation between microvascular changes in macula in OCTA and peripheral ischemia [7, 8]. The correlation between peripheral ischemia with vascular density in superficial and deep capillary plexuses in patients with retinal vein occlusion was documented previously [7]. The vascular density less than 46% was significantly associated with peripheral ischemia as capillary non perfusion more than one quadrant in fluorescein angiography [7]. On the other hands, the amount of vascular density may be related to severity of retinal ischemia [7]. Coscas et al. [8] reported the correlation between the disruption in perifoveal capillaries in OCTA and peripheral ischemia in fluorescein angiography. Mastropasqua et al. [5] founded lower amounts of vascular density in 3 main capillary plexuses including superficial and deep capillary plexuses and choriocapillaris were associated with retinal ischemia at fluorescein angiography [5]. It can be concluded that the parameters of OCTA may help as factors in differentiation presumably ischemic CRVO from non ischemic CRVO. To best of our knowledge this is the first report that compares the OCTA parameters between ischemic CRVO and non ischemic CRVO and introduces a formula by using OCTA parameters as discrimination factor in addition to previous factors including size of retinal ischemia in fluorescein angiography, electroretinogram findings and etc. in diagnosis of retinal ischemia. In this study we found the reduced formula (3.9 × F_1S_ + 0.8 × F_3S_) by choosing a threshold of 12.6 (ischemic type < 12.6) can recognize the ischemic type from non ischemic CRVO with the AUC of 0.84 and accuracy of 96%. It can be concluded that OCTA at least helps in diagnosis of CRVO type that requires fluorescein angiography.

For finding the most damaged parameter we utilized the mean ratio. The mean ratio of flow in deep plexus of macular area was lower significantly than other parameters. Similar to our result, the previous studies reported the deep capillary plexus was more influenced in retinal vein occlusion than superficial plexus [5, 8, 15]. Coscas et al. [8] assessed the OCTA in patients with retinal vein occlusion. They reported the capillary plexuses abnormalities were significantly more common in deep plexus than superficial plexus. They concluded that deep capillary plexus was influenced more severely in retinal vein occlusion. The higher amount of cystoids spaces and resultant disorganization of deep plexus in comparison to superficial plexus was accounted as possible explanation of this finding [8, 10]. They also related lesser involvement and better perfusion of superficial plexus owing to direct connection to retinal arterioles in contrast to deep capillary plexus [8]. They also found OCTA can detect macular edema better than spectral domain OCT and fluorescein angiography [8]. The perfusion in deep capillary plexus was influenced severely and more rapidly as result of augmentation intravascular pressure and hydrostatic pressure owing to connection to major veins in retinal vein occlusion [16]. The location of deep plexus is coincide with watershed area that leading to be vulnerable this capillary plexus to ischemic insult [17].

We observed significant correlation between visual acuity and all parameters of OCTA. The most significant correlation was found between the flow in choriocapillaris and visual acuity. It may be related to severity of the disease. On the other hands, the severity of the choriocapillaris might directly relate to severity of CRVO and its ischemia. So, the more involvement of choriocapillaris associated with lower visual acuity. Mastropasqua et al. [5] found the functional parameters like visual acuity was not correlated with vascular density in OCTA in contrast to our results. The difference of severity of their patients in comparison to our patients may partly explain this discrepancy. The other studies reported the similar results like our results [7, 15, 18]. Seknazi et al. [7] reported significant correlation between vascular density and visual acuity. The visual acuity (LogMAR) correlated reversely with vascular density in superficial and deep plexuses in patients with BRVO in the study of Samara et al. [18]. Vascular perfusion in deep plexus was reported that correlated with visual acuity in patients with BRVO as the most significant parameter [15]. The OCTA may be used as a prognostic factor in visual outcome.

Our investigation had some limitations. OCTA cannot be used in patients with significant opacity and poor fixation so some of our patients excluded. Limited sample size was another limitation of our study and future studies with more patients are needed to establish our finding. Our patients had acute CRVO so the assessment of accurate ischemic area in retinal periphery was difficult using fluorescein angiography owing to retinal hemorrhage. So the correlation between parameters of OCTA with amount of peripheral ischemia could not be evaluated. Severe macular edema had some degrees of interfering with accurate interpretation of OCTA parameter due to probable signal attenuation and disorganization of structures leading to difficulty precise distinguish of superficial plexus from deep plexus. The automated segmentation in our instrument can cause some limits in selection of patients and interpretation of images due to artifacts. Due to these 2 reasons, we had to exclude some of our patients with severe macular edema. So, our results generalized only to selected patients with CRVO not to all patients (with severe macular edema) and this was one of the major limitation of our study.

## Conclusion

The OCTA can accurately evaluate microvascular structures of retina and choriocapillaris in patients with CRVO. As previously reported, the deep plexus was influenced more severe than superficial plexus and choriocapillaris. The worth of information on vascular flow in three main capillary plexuses provided by OCTA is so valuable, which makes it helpful for recognition of microvascular abnormalities in retinal vein occlusion and quantifies the degree of severity of CRVO. OCTA may have a prognostic value in visual outcome and in distinguishing presumably ischemic CRVO from non ischemic CRVO. A future prospective, randomized, controlled study with a large sample size is needed to confirm the current results.
